# Heat and Moisture Exchanger Occlusion Leading to Sudden Increased Airway Pressure: A Case Report Using ChatGPT as a Personal Writing Assistant

**DOI:** 10.7759/cureus.37306

**Published:** 2023-04-08

**Authors:** Alejandro Hallo-Carrasco, Benjamin F Gruenbaum, Shaun E Gruenbaum

**Affiliations:** 1 Department of Pain Medicine, Mayo Clinic, Jacksonville, USA; 2 Department of Anesthesiology and Perioperative Medicine, Mayo Clinic, Jacksonville, USA

**Keywords:** chatgpt, intubation complication, prolonged ventilation, hme, humidified moisture exchanger

## Abstract

Heat and moisture exchangers (HMEs) are commonly used during general anesthesia to provide appropriate humidification and warming of inspired gases. While they play a critical role in mechanical ventilation, they can also lead to acute difficult ventilation if not correctly monitored and drained. We present a case of a 56-year-old female patient who underwent lower extremity vascular bypass surgery under general anesthesia and experienced sudden increased airway pressures due to occlusion of the HME caused by excessive moisture accumulation. Proper monitoring and management of the airway circuit and HMEs can help prevent complications and ensure proper ventilation during surgery. When acute difficult ventilation is encountered during general anesthesia, a systematic approach should be taken to differentiate between patient and external factors. Other differential diagnoses for acute difficult ventilation include bronchospasm, aspiration, endotracheal tube misplacement, pulmonary embolism, and tension pneumothorax. HME occlusion should be considered as part of the differential diagnosis for intraoperative hypoxia. Proactive replacement of HMEs in long cases can prevent occlusion and ensure proper ventilation.

## Introduction

Acute hypoxia with increased airway pressures during general anesthesia is a common problem that requires prompt diagnosis and intervention. While patient factors such as bronchospasm, aspiration, and endotracheal tube misplacement are often the first considerations, problems with the airway circuit and heat and moisture exchangers (HMEs) can also lead to acute difficult ventilation [1,2].

HMEs are commonly used in anesthesiology to provide with appropriate humidification as well as warming of inspired gases during intubation and maintain the heat and moisture properties of the upper respiratory tract [3]. HMEs are placed between the breathing circuit and proximally to the endotracheal tube, where they humidify and warm inspired gases. While they play a crucial role in mechanical ventilation, they also have some potential risks [4,5].

We will explore the risk of occlusion of the HME with a case of sudden increased airway pressure during a lower extremity vascular bypass surgery under general anesthesia.

## Case presentation

A 56-year-old female patient with coronary artery disease status post percutaneous coronary intervention and ischemic systolic heart failure (ejection fraction (EF) 20%), hypertension, stage III chronic kidney disease, and a previous stroke underwent a left femoral to posterior tibial bypass surgery under general anesthesia. The patient was intubated with an endotracheal tube and connected to a breathing circuit with an HME to provide appropriate humidification and warming of inspired gases during the surgery. The patient was maintained under general anesthesia with sevoflurane (0.5-1.0 minimum alveolar concentration (MAC)), and muscle relaxation was achieved with rocuronium. Both sevoflurane and rocuronium were used for the duration of the procedure.

**Figure 1:**
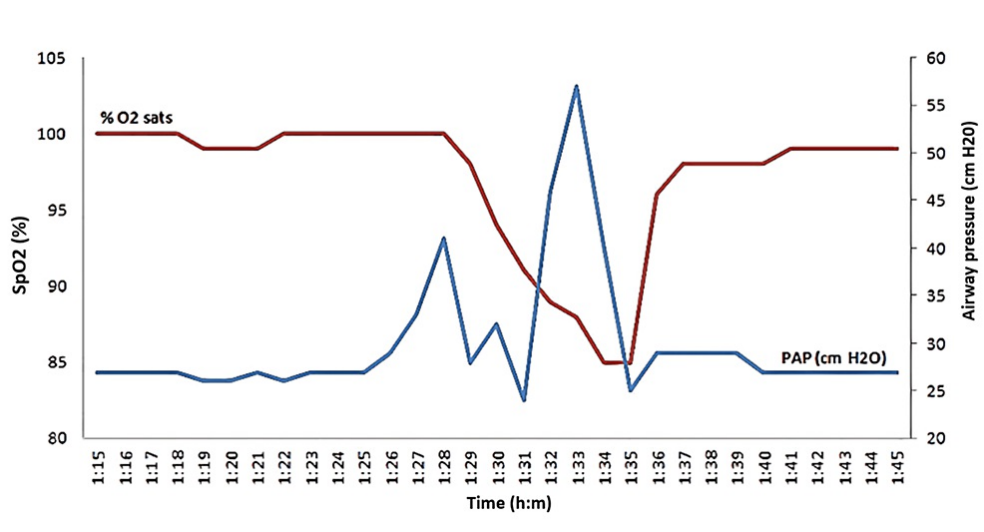
Desaturation with increased positive airway pressure over time SpO2: oxygen saturation; %O2 sats: % of oxygen saturation; PAP: peak airway pressure

Twelve hours after the initial incision, the patient suddenly experienced a significant increase in peak airway pressures from 27 to 41 mmHg, decreased tidal volumes from 500 to 70 cc, and desaturation from 100% to 85% (Figure 1). The patient’s ventilation mode was switched from volume control to spontaneous ventilation to evaluate for ventilator dyssynchrony. The fraction of inspired oxygen (FiO2) was increased from 0.5 to 1.0. The anesthesiology team aggressively suctioned the airway, and albuterol (approximately 10 puffs) was administered down the endotracheal tube. Despite these interventions, there was no observed improvement in her condition. The endotracheal tube was disconnected from the circuit at the connection between the tube and HME. The HME used was a sterile, single-use, latex-free, high filtration efficiency HME with an electrostatic membrane filter (Teleflex 1589 Hudson RCI; Teleflex Inc., Wayne, Pennsylvania, United States). A large amount of thick moisture was observed in the HME filter, and upon squeezing the reservoir bag with the adjustable pressure limiting (APL) valve closed, the moisture was expelled from the HME. The circuit was then reconnected, and the patient was easily ventilated thereafter with complete resolution of the increased airway pressures. The patient's oxygen saturation quickly increased to 100%. The procedure was completed, and the patient was extubated with no further complications.

## Discussion

HMEs are commonly used during general anesthesia to provide humidification and warming of inspired gases, which can help prevent airway irritation and injury. However, their use can also lead to complications such as HME occlusion, disconnections, unnoticed leaks, and risk of infections, which can result in an acute increase in airway pressure and difficulties with ventilation. HME occlusion can occur due to a variety of factors, including excessive moisture accumulation from the patient's exhalation or the breathing system, kinking of the HME tubing, or blockage of the HME filter [3,6]. In our case, the accumulated moisture was likely the result of a buildup of condensed exhaled gases, which had clogged the HME’s electrostatic membrane filter.

**Figure 2:**
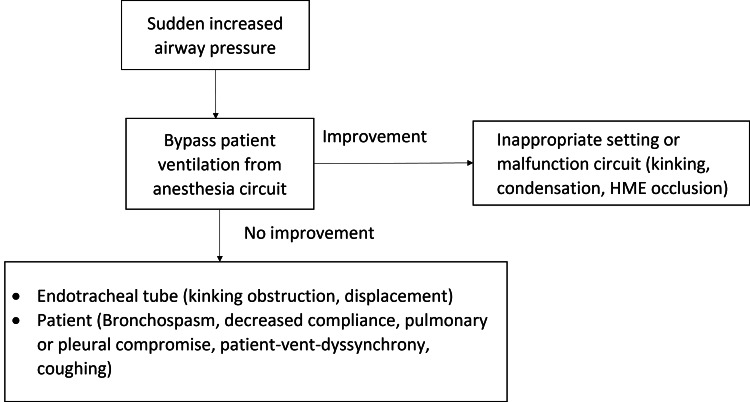
Algorithm for sudden increase in positive airway pressure HME: heat and moisture exchanger

Proper monitoring and drainage of the airway circuit and HMEs are important to prevent complications and ensure proper ventilation during surgery. In cases of acute difficult ventilation during general anesthesia, a systematic approach should be taken to differentiate between patient and external factors. One useful tool to differentiate patient factors from external factors is a flowchart (Figure 2). The first step in the flowchart is to connect the endotracheal tube to an Ambu bag and see if that improves the airway pressures [7]. If the airway pressures improve, it suggests that the problem is external to the patient and likely related to the airway circuit or HME. If the airway pressures do not improve, it suggests that the problem is likely related to patient factors such as bronchospasm or endotracheal tube misplacement. Other diagnostic tests such as chest X-rays, blood gas analysis, and bronchoscopy may also be useful in identifying the cause of acute difficult ventilation.

While HME occlusion is a rare cause of intraoperative hypoxia, it should be considered as part of the differential diagnosis. In our case, other differential diagnoses were also considered. Pneumothorax was unlikely due to the nature of the procedure and a lack of other supporting evidence (such as unilateral decreased breath sounds). Anaphylaxis was also considered; however, there were no observable signs of any skin rash or changes in blood pressure or heart rate, and no new medications were recently administered. Bronchospasm and mucus plugging were high on the differential diagnosis, and these were quickly addressed with bronchodilator administration and aggressive suctioning, respectively, without any resolution of the increased airway pressures. Ventilator dyssynchrony was also ruled out by taking the patient off the ventilator and ensuring adequate muscle relaxation. Other differential diagnoses for acute difficult ventilation include bronchospasm, aspiration, endotracheal tube misplacement, pulmonary embolism, and tension pneumothorax. Proper monitoring and management of the airway circuit and HMEs can help prevent complications and ensure proper ventilation during surgery, and excessive manipulation of the patient's airway can be minimized [1,2].

Teaching points

When a sudden increase in peak airway pressure and difficulties in ventilation are encountered, one should consider etiologies intrinsic and extrinsic to the patient. An HME occluded with condensed moisture is a rare but important cause of sudden increase in peak airway pressures and difficulty in ventilation, particularly in long cases. In long cases in which an HME is used, one should consider replacing the HME to prevent it from becoming occluded with secretions and moisture.

ChatGPT considerations

For this scientific paper, we employed ChatGPT 3.5 (OpenAI, San Francisco, California, United States) as a personal writing assistant to improve the coherency and clarity of our ideas (See Appendix). We utilized its capabilities to remove redundancies and streamline our writing process. Artificial intelligence (AI) is an excellent resource for academic writers, but it cannot replace the essential human element of writing a paper.

ChatGPT focuses on structured and straightforward writing without considering the hypotheses, suspicions, and nuances that are fundamental in academic writing. Therefore, using AI alone may limit the paper's ability to explore future research directions or potential implications fully. It is important for writers who are using ChatGPT as a tool to enhance their writing, to incorporate their own insights and knowledge, develop hypotheses, and add coherency to the results in the paper to ensure a well-rounded and comprehensive final product [8].

While AI is a powerful language model with a vast amount of knowledge, it currently does not have the ability to provide valid citations or high-quality sources. In fact, there have been instances where ChatGPT has generated inexistent references or added Digital Object Identifiers (DOIs) from fields outside of medicine, which can potentially lead to misinformation if not recognized and addressed by an expert on the topic [9]. As such, it is important for users of AIs to exercise caution and critical thinking when using the information provided by the AI [10]. The lack of adequate citation mandates users to have a strong understanding of the topic at hand and to seek out additional sources of information to verify the accuracy of ChatGPT's responses.

Despite these limitations, our approach to utilizing ChatGPT as a writing assistant ensured that authors added only high-quality, relevant information to the scientific literature. We carefully reviewed and fact-checked all content generated by ChatGPT to ensure its accuracy before incorporating it into our paper.

Future directions

We strongly encourage authors to consider the potential applications of AI, such as in education and research, and to explore these areas through well-defined projects. However, we want to emphasize that ChatGPT is currently in beta mode, and caution should be exercised when interpreting its responses. Some research questions that could be addressed in future projects include investigating the impact of ChatGPT on existing literature and on different types of users, examining differences between ChatGPT statements depending on the user's level of expertise, or exploring the potential of this AI as a tutor for novice authors.

## Conclusions

HME occlusion is a rare but important cause of intraoperative hypoxia that should be considered as part of the differential diagnosis in cases of acute difficult ventilation during general anesthesia. A systematic approach, including the use of diagnostic tools such as flowcharts and diagnostic tests, is important to differentiate between patient and external factors and identify the cause of acute difficult ventilation. Proper monitoring and management of the airway circuit and HMEs can help prevent complications and ensure proper ventilation during surgery.

## Figures and Tables

**Figure 3:**
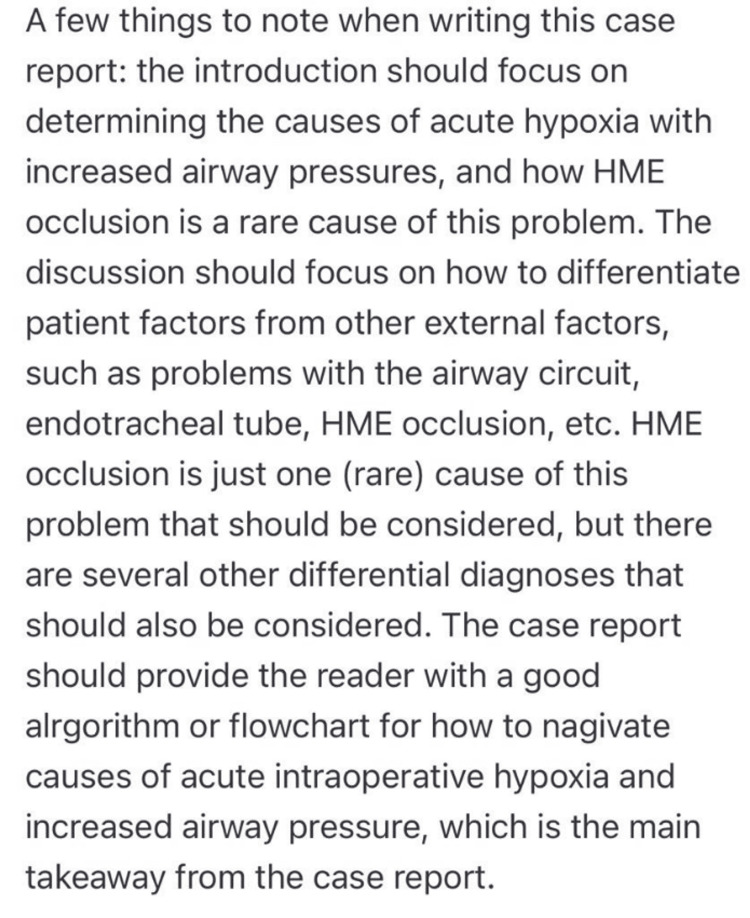
Answer of ChatGPT to “Can you write a case report for the journal Cureus? Here are the details of the case:…..”

**Figure 4:**
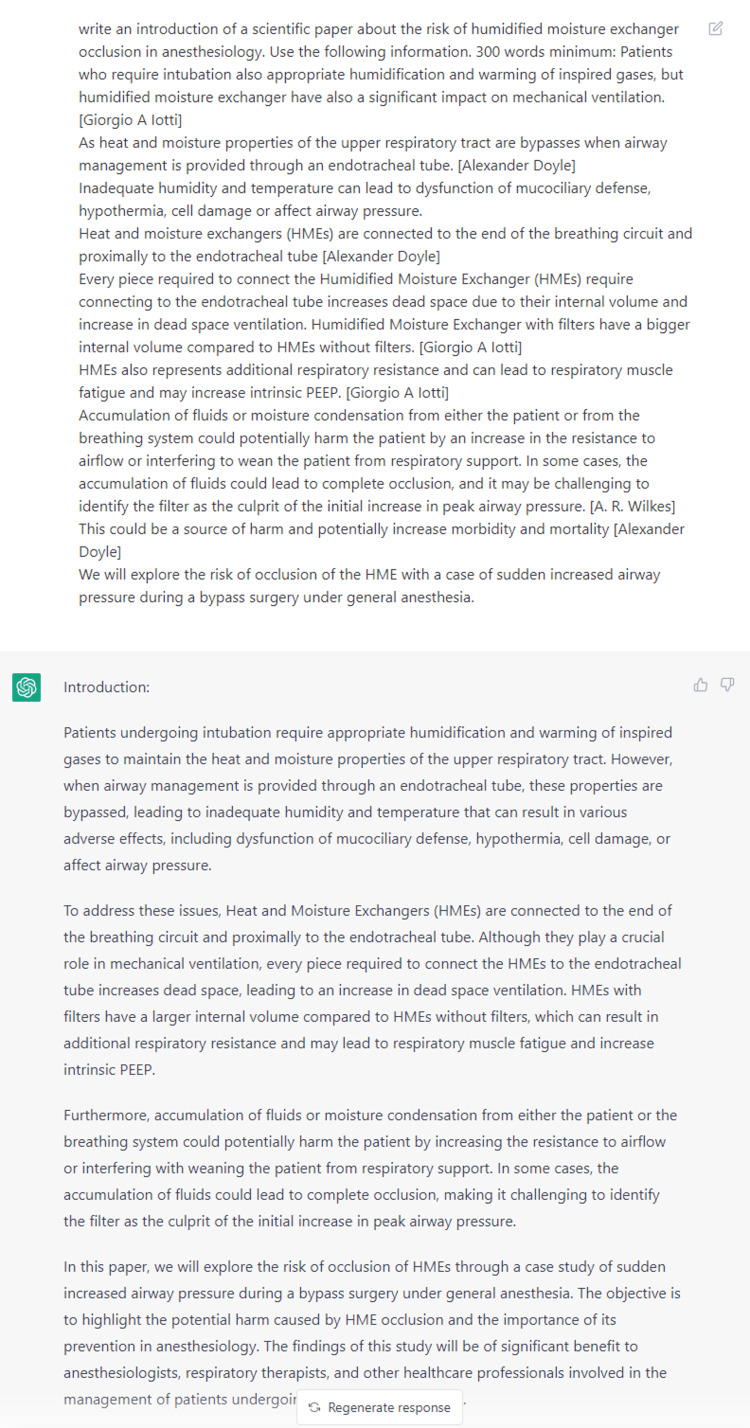
Asking ChatGPT to write an introduction based on information provided

**Figure 5:**
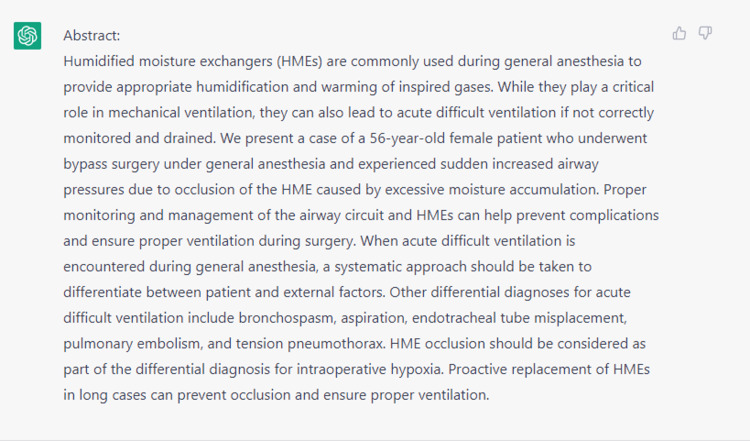
Asking ChatGPT to write an introduction based on information provided
